# Teamwork and Adherence to Guideline on Newborn Resuscitation—Video Review of Neonatal Interdisciplinary Teams

**DOI:** 10.3389/fped.2022.828297

**Published:** 2022-02-21

**Authors:** Lise Brogaard, Lone Hvidman, Gitte Esberg, Neil Finer, Kristiane R. Hjorth-Hansen, Tanja Manser, Ole Kierkegaard, Niels Uldbjerg, Tine B. Henriksen

**Affiliations:** ^1^Department of Obstetrics and Gynecology, Aarhus University Hospital, Aarhus, Denmark; ^2^Department of Clinical Medicine, Aarhus University, Aarhus, Denmark; ^3^Department of Pediatrics, Aarhus University Hospital, Aarhus, Denmark; ^4^Department of Neonatology, University of California, San Diego, San Diego, CA, United States; ^5^Department of Obstetrics and Gynecology, Aalborg University Hospital, Aalborg, Denmark; ^6^School of Applied Psychology, University of Applied Sciences and Arts Northwestern Switzerland, Olten, Switzerland; ^7^Department of Obstetrics and Gynecology, Horsens Regional Hospital, Horsens, Denmark

**Keywords:** neonatal resuscitation, patient safety, quality improvement, video, teamwork

## Abstract

**Background:**

Little is known about the importance of non-technical skills for the adherence to guidelines, when teams of midwives, obstetricians, anesthesiologists, and pediatricians resuscitate and support the transition of newborns. Non-technical skills are competences underpinning successful teamwork in healthcare. These are usually referred to as leadership, situational awareness, communication, teamwork, decision making, and coping with stress and fatigue.

**Objective:**

By review of videos of teams managing newborns with difficult transition, we aimed to investigate whether the level of the teams' non-technical skills was associated with the degree of adherence to guidelines for newborn resuscitation and transitional support at birth.

**Methods:**

Four expert raters independently assessed 43 real-life videos of teams managing newborns with transitional difficulties, two assessed the non-technical score and two assessed the clinical performance. Exposure was the non-technical score, obtained by the Global Assessment Of Team Performance checklist (GAOTP). GAOTP was rated on a Likert Scale 1–5 (*1* = poor, *3* = average and *5* = excellent). The outcome was the clinical performance score of the team assessed according to adherence of the European Resuscitation Counsel (ERC) guideline for neonatal resuscitation and transitional support. The ERC guideline was adapted into the checklist TeamOBS-Newborn to facilitate a structured and simple performance assessment (low score 0–60, average 60–84, high 85–100). Interrater agreement was analyzed by intraclass correlation (ICC), Bland-Altman analysis, and Cohen's kappa weighted. The risk of high and low clinical performance was analyzed on the logit scale to meet the assumptions of normality and constant standard deviation.

**Results:**

Teams with an excellent non-technical score had a relative risk 5.5 [95% confidence interval (CI) 2.4–22.5] of high clinical performance score compared to teams with average non-technical score. In addition, we found a dose response like association. The specific non-technical skills associated with the highest degree of adherence to guidelines were leadership and teamwork, coping with stress and fatigue, and communication with parents. Inter-rater agreement was high; raters assessing non-technical skills had an interclass coefficient (ICC) 0.88 (95% CI 0.79–0.94); the neonatologists assessing clinical performance had an ICC of 0.81 (95% CI 0.66–0.89).

**Conclusion:**

Teams with an excellent non-technical score had five times the chance of high clinical performance compared to teams with average non-technical skills. High performance teams were characterized by good leadership and teamwork, coping with stress, and fatigue and communication with parents.

## Introduction

The first breath of a newborn initiates the transition from placental dependence to independent life (1). An unsuccessful transition is a high-risk situation and is a significant part of the 2.7 million neonatal deaths per year (2). Improving neonatal care is a global priority and even in high income countries the support of newborns can be suboptimal (3).

The skills to support transition and resuscitate the newborn are essential for every healthcare provider working in the delivery ward. Most newborns adapt well to life after birth. However, some (5–10%) need positive pressure ventilation but only few (0.1–0.3%) receive chest compressions (4). A prerequisite for successful neonatal resuscitation is prompt recognition of signs of a difficult transition and a rapid and relevant coordinated response by the team (5). As the use of more than a few steps in the algorithm of neonatal resuscitation is rare, it is a challenge for healthcare providers to become experienced and deliver a coordinated response. The competences underpinning successful teamwork in healthcare are cognitive, social and personal resources usually referred to as non-technical skills. These skills are described as leadership, situational awareness, communication, teamwork, decision making, and coping with stress and fatigue (6).

All resuscitation teams try to follow current guidelines to produce the best infant outcomes, but performance may fall short of expectations (7). As a result, there is a need for evaluation and educational strategies which include simulation-based training, audit, feedback, and debriefing all of which are facilitated using video review. Little is known about the importance of non-technical skills and interdisciplinary teamwork of resuscitation teams. Video recording such teams (2) to review their performance enables in-depth analysis of non-technical and clinical team performance (1, 8–10). Prediction of a problematic transition and the need for resuscitation may be difficult and as a result, first line personnel including midwives and obstetricians may need to provide resuscitative interventions in the first minutes after birth.

The purpose of this study was to utilize video recording in the delivery rooms to investigate whether the level of non-technical skills in interdisciplinary teams of midwives, obstetricians, anesthesiologists, and pediatricians was associated with the degree of adherence to newborn resuscitation guidelines.

## Materials and Methods

### Study Design and Setting

Real-life video-recordings of newborns were collected from November 2014 to February 2016 at two Danish hospitals; Aarhus University Hospital and Horsens Regional Hospital. Aarhus University Hospital, with ~5,000 deliveries per year, provides maternal care level III (11) and neonatal care level III (12). Horsens Regional Hospital, with ~2,000 deliveries per year, provides level II maternal care, and has no pediatric service in the Hospital, but anesthesiologists and obstetricians are trained to provide advanced neonatal life support and work in close collaboration with the neonatal transport team from Aarhus University Hospital who will provide advice and who will send an expert neonatal transport team if needed. The fastest arrival is within 30–45 min after the request.

All 17 delivery suites at the two hospitals were equipped with two or three high-definition mini-dome surveillance cameras and a microphone in the ceiling to cover an oblique view of the room, and the neonatal resuscitation table. Recordings were automatically activated by blue tooth as described previously (13). In case the pregnant woman declined participation no recordings were made. Inclusion criteria were: vaginal birth of a newborn with insufficient response to stimulation, gestational age 35 weeks or more. Duration of the videos included was 9 min. We started the video 2 min before delivery to ensure that raters could assess any preparation before delivery. It was ended seven minutes after delivery no matter how long the video was to minimize potential bias by a longer videoclip, which would allow for altered performance and change of team members. Video was started 2 min before because of maternal problems.

### Video Analysis of Non-technical Score

Exposure was the non-technical score obtained by the Global Assessment Of Team Performance checklist (GAOTP) developed by Morgan et al. (14, 15). The GAOTP checklist was selected among several validated tools (16), as it was developed and validated for assessment of non-technical performance of interdisciplinary teams in the delivery room.

The checklist included six dimensions:

1) *Communication with patient/parents*: Excellent team performance was defined as sharing information with the parents and when possible, involving parents in the newborns care.

2) *Task/case management*: excellent team performance was scored if urgency of the clinical situation was recognized, goals were set and communicated with the team and resources were effectively utilized.

3) *Leadership and teamwork*: excellent team performance was scored if roles were quickly established, a leader was identified, leader encouraged participation and identified opportunities for improvement, and team's roles, and responsibilities were clear.

4) *Situational awareness*: excellent team performance was scored if there was early recognition and a rapid response to a critical situation, if extra personnel were summoned in a timely fashion, and team members remained vigilant, and alert to the clinical situation.

5) *Communication with team members*: excellent team performance was scored if communication was focused with clear questions and instructions directed to a specific person, receiver acknowledged receipt of message and confirmation that requested actions was completed.

6) *Environment of the room*: excellent team performance was scored if the environment was orderly and controlled, voices remained calm and focused, no sign of stress or fatigue among team members.

Each of the six dimensions were rated by a Likert scale 1–5. GAOTP overall score was the mean score on a Likert scale, where “*1*” was a poor non-technical score, “*3*” was an average non-technical score, and “*5*” was an excellent non-technical score (Supplementary Material 1 Description of GAOTP dimensions). Two physicians (authors LB, KRH) experienced in the use of GAOTP assessed all the videos using the GAOTP checklist. All videos were rated by both reviewers blinded to the other reviewer's ratings (13).

### Video Analysis of Clinical Performance

The primary outcome was the teams' clinical performance score assessed according to adherence of the European Resuscitation Counsel (ERC) guideline for neonatal resuscitation and transitional support. In both hospitals all healthcare providers in the Labor and Delivery units (midwives, obstetricians, anesthesiologists, and pediatricians) were trained by the ERC guideline. As the guideline was updated during the study, both the more recent and the previous guideline was accepted as part of our checklist, e.g., in the assessment of heartrate we accepted any assessment as there was no specific recommendation for heart rate measurement in the 2010 guideline and the 2015 guideline recommended this be changed to a 3-led electrocardiogram. The ERC guideline was adapted into the checklist '*TeamOBS-Newborn'* to facilitate a structured and simple performance assessment (17, 18). The checklist TeamOBS-Newborn consisted of seven categories each with a number of items to be ticked off. The Categories were: (1) *Preparation of equipment and room*, 9 items; (2) *Initial assessment and support of transition*, 7 items; (3) *A-problems*, 5 items; (4) *B-problems*, 4 items; (5) *C-problems*, 4 items; and (6) *Global score*. The raters assessed all items according to the ERC guideline in three levels: 0 points (not done or done incorrectly), 1 point (done partially or not done in a timely manner) and 2 points (done correctly and in a timely manner). We also included the category *Cannot be assessed* or *not indicated* (e.g., if an IV line was not indicated) (no value). The score of each item was then multiplied by the assigned weight of the item and totaled to give a weighted score, i.e., a percentage of the highest possible score [a maximum of 58 if all items were assessed, but less if one or more items were marked as *Cannot be assessed* or *Not indicated*]. To assess 'high clinical performance' we used the ERC guideline (“checklist”) as one of two parts of the evaluation. The second part of the evaluation was a global score of patient safety introduced in order to allow the experienced raters to provide the team with a numeric score from 0 to 100 on a visual analog scale; with 100 reflecting best expert standard. The TeamOBS-newborn tool resulted in a clinical performance score from 0 to 100, where low clinical performance was 0–59, average clinical performance was 60–84, and high clinical performance was 85–100 (Figure S1). Two experts in neonatology (TBH and GE) assessed all videos independently by use of the checklist and were blinded for each other's ratings.

None of the raters were involved in the delivery or the management of the newborn.

### Ethics and Legal Aspects

All videos were included following informed consent in conformity with the Danish penal code §264. Gaining informed consent during labor was not acceptable according to the Danish Health Act §43–44. Women were informed about the research project TeamOBS during antenatal visits. All women who planned to give birth in one of the two Labor and Delivery wards were informed between gestational week 18–28 by oral and written information provided by a sonographer or a midwife. Whether the woman accepted or denied participation was noted in her medical record. If participation was declined cameras were inactivated. If participation was accepted the cameras were activated automatically by staff's mobile devices if resuscitation of the newborn was initiated. The use of any recording should be approved within 48 h after delivery by all persons present in the room; i.e., the woman giving birth, her husband or any other person she had asked to be present, and all healthcare providers. If written consent was missing from one person the video was automatically deleted. The research project TeamOBS was approved legally and ethically in May 2014 by the Central Denmark Region, the Danish Data Protection Agency (2012-58-006) and the Research foundations of central Denmark (1-16-02-257-14).

### Statistical Analysis

Agreement among raters was identified by the intraclass correlation (ICC) and Bland-Altman analysis (19). The non-technical score was also analyzed by category by Cohen's kappa weighted and percentage agreement (20). The association between the non-technical score and clinical performance was described using a restricted cubic spline regression analysis with three knots at 2.5, 3, and 3.5 on the non-technical score (21). The mean difference and relative risk in clinical performance between the lowest “*3*” and the highest non-technical score “*5*” was estimated using spline regression analysis. The risk of high and low clinical performance was analyzed on the logit scale to meet the assumptions of normality and constant standard deviation (22). 95% confidence intervals (CI) were calculated by non-parametric percentile bootstrap. Regression models were checked using diagnostic plots of residuals, and two-sided *p* < 5% were considered statistically significant. We conducted all statistical analysis by STATA 15 (Stata Corp LP, College Station, TX, USA).

## Results

### Included Videos

From November 2014 to February 2016, we informed all women, expecting to give birth at one of the study hospitals about the research project. Consent was declined by few healthcare providers, mothers, or relatives. We included 43 videos of teams managing newborns. Of these, 58% of the newborns received continuous positive airway pressure (CPAP), 28% received five inflations breaths, 23% were suctioned, and 2% received chest compressions and 42% needed no further intervention.

### Inter-rater Agreement

Raters assessing non-technical skills achieved an ICC of 0.88 (95% CI 0.79–0.94); the neonatologists assessing clinical performance achieved an ICC of 0.81 (95% CI 0.66–0.90), Table 1. Agreement among raters assessing non-technical skills, on single item level, was 0.82–0.90 weighted Kappa and agreement was visualized by Bland Altman Plots and limits of agreement (Table S1, Figure S2).

**Table 1:** Inter-rater agreement for clinical performance and non-technical performance.

	**Descriptive**		**ICC (95% CI)**			
	**Mean**	**Range**	Individual rater^*^		Average of two raters^**^	
Non-technical score GAOTP	23	(12–30)	0.79	(0.65–0.88)	0.88	(0.79–0.94)
Clinical performance: TeamOBS-newborn	81	(28–100)	0.69	(0.49–0.82)	0.81	(0.66–0.90)

* *Intraclass correlation (ICC) between one rater and the other*.

** *All 43 videos were analyzed by 4 raters, 2 for non-technical, and 2 for clinical performance*.

### Non-technical and Clinical Performance

**Figure 1:**
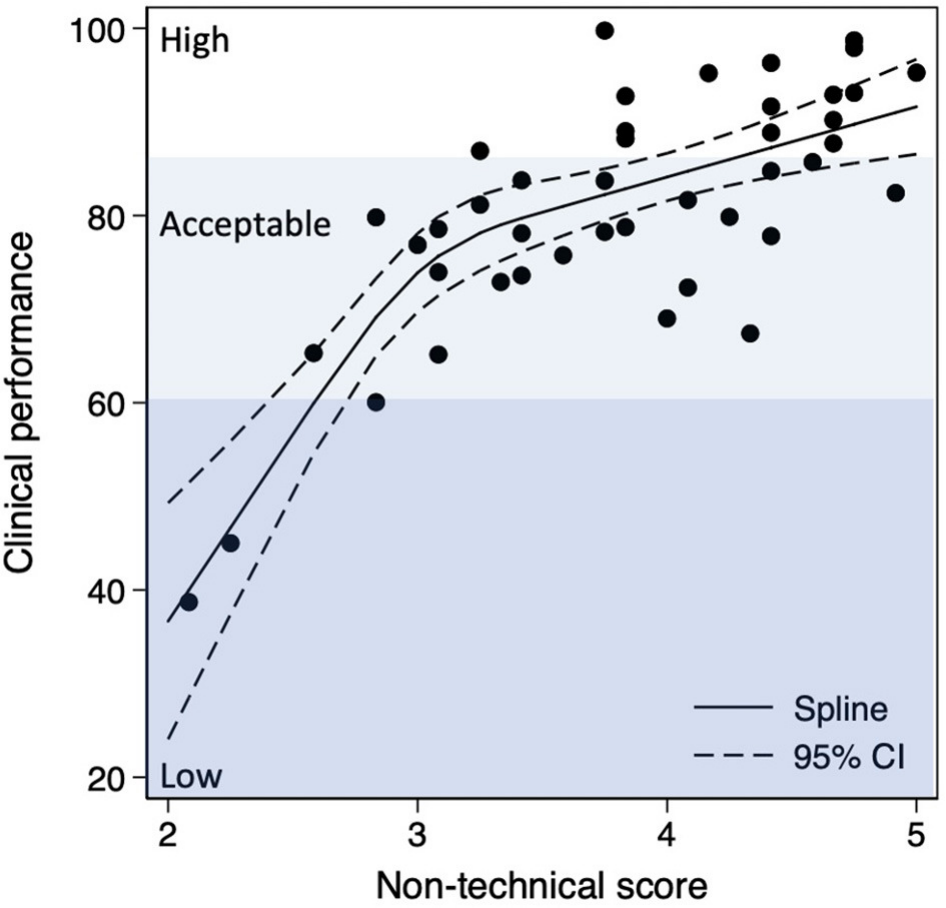
Non-technical score and clinical performance is visualized by spline regression analysis.

Teams with low and average clinical performance (clinical performance <85) often had a lower score due to the use of CPAP to a newborn who was judged by the experts to have no need or due to delayed assessment of the newborn's heartrate. The clinical performances and the non-technical score were correlated and showed a dose-response like association (Figure 1). Thus, an excellent non-technical score was associated with a high clinical performance (adherence to guideline score ≥ 85) of 86.5% (95% CI 68.7–97.5%) compared with 15.6% (95% CI 3.8–30.7%) for an average non-technical score (Table 2). This corresponded to a RR 5.5 (95% CI 2.4–22.5) of high clinical performance in teams with an excellent compared to an average non-technical score.

**Table 2:** Chance of clinical performance according to the level of non-technical performance.

**Non-technical score**	Risk of low clinical performance (95% CI)^*^		Chance of high clinical performance (95% CI)^**^		RR for high clinical performance (95% CI)^****^	
2 (poor) (%)^***^	94.4	(47.3–99.6)	0.01	(<0.01–2.5)		
3 (average) (%)	14.7	(6.2–25.2)	15.6	(3.8–30.7)		
4 (good) (%)	1.4	(0.1–3.7)	54.8	(40.6–66.7)	1.5	(1.3–2.0)
5 (excellent) (%)	<0.01	(<0.01–1.0)	86.5	(68.7–97.5)	5.5	(2.4–22.5)

* *TeamOBS-newborn score below minimal pass (score > 60%)*.

** *TeamOBS-newborn score above high performance (score ≥ 85%)*.

*** *Limited data for the score of 2 (see Figure 1)*.

**** *Reference group average (3) non-technical score*.

High performing teams demonstrated excellent team behavior in the non-technical dimensions: leadership and teamwork, coping with stress and fatigue, and communication with the newborn's parents. The mean differences are listed in Table 3 and the association of individual non-technical categories and clinical performance are visualized in Figure S3. There was no significant difference in teams' clinical performance between the two hospitals and no differences by time of day (day shift, evening shift, and night shift).

**Table 3:** Mean difference in clinical performance between non-technical score 5–3.

	**Non-technical score (3)**		**Non-technical score (5)**		**Mean difference**	
	**Mean**	**95% CI**	**Mean**	**95% CI**	**Diff**	**95% CI**
1. Communication with parents	75.9	(66.4–83.7)	88.1	(83.3–91.8)	12.1	(1.9–22.4)
2. Task management	82.5	(72.3–72.3)	87.7	(82.2–92.0)	5.3	(−5.4–15.9)
3. Leadership and teamwork	82.0	(76.1–86.9)	93.0	(87.2–96.8)	11.0	(3.0–19.0)
4. Situational awareness	77.9	(62.8–88.7)	85.8	(80.9–89.8)	7.9	(−6.1–21.9)
5. Communication with team members	81.5	(74.3–97.3)	90.3	(83.8–94.9)	8.9	(−0.7–18.4)
6. Environment, coping with stress, and fatigue	69.3	(58.0–79.0)	90.7	(87.4–93.4)	21.4	(9.8–33.0)
Summative non-technical score (GAOTP)	74.3	(67.3–80.4)	92.8	(88.7–95.9)	18.5	(10.0–27.0)

## Discussion

Teams with an excellent non-technical score had five times more likely to have a high clinical performance than teams with an average non-technical score. The clinical performance and the non-technical score were strongly correlated with a dose-response like association. High performing teams demonstrated excellent team behavior in leadership and teamwork, coping with stress and fatigue, and communication with parents.

The main strength of this study was that evaluation of the management of difficult transition was based on real-life video recordings of teams of midwives, obstetricians, anesthesiologists, and pediatricians managing newborns. The technical solution was also a strength as our automated activation of cameras ensured enrolment of videos both day and night. Another strength was the systematic video analyses as four trained raters assessed all videos independently by use of structured and validated checklists and a high interrater agreement (23).

The study was somewhat limited by the small number of videos, which reduced the possibility of performing sub-analyses, e.g., related to team construction, or level of newborn life support needed. However, to optimize learning from this study we insured that only newborns thought to be in need of transitional support or resuscitation participated in the study. We believe, that our study population corresponded to a random sample of the entire population of newborns with similar needs.

Concerning the internal validity, we cannot exclude that teams that perceived their own performance as suboptimal were more prone to deny participation. Furthermore, the TeamOBS-newborn was not validated *a priori*. However, it was developed by stringent use of the detailed version of the current ERC guideline and the inter-rater agreement was high (24). Furthermore, it was impossible to blind raters of the clinical performance to the team's non-technical score and vice versa. However, the use of several raters and checklists limited this problem, and all raters were blinded to the other raters scores. Finally, our study found that clinical performance and the non-technical score was strongly correlated with a dose-response like association. However, this is not a proof of causality.

This study and prior studies demonstrated that video can capture teams' behavior, timing of procedures, and the flow of an algorithm and can be reviewed repeatedly (8). This information can be difficult for the healthcare providers to recall after the management of an emergency, and studies have found written documentation in the patient files may be misleading when compared to videos (25, 26). However, due to the requirements of informed consent and legal concerns, videos are used rather rarely in clinical practice even though it was already introduced in 1969 (27–29). We addressed this challenge by informing all women who planned a vaginal birth before delivery.

Research in non-technical skills described as leadership, situation awareness, communication, teamwork, decision making and coping with stress and fatigue have revealed that the importance of these specific skills differs among settings, tasks and specialities e.g. between *ad hoc* teams for resuscitation and surgical teams conducting planned procedures (30–32). Thus, team communication, task management and leadership were fundamental for team adherence to guidelines in a setting where pediatric teams managed 132 newborns delivered by planned cesarean section (10). The majority of these newborns were healthy. A sub-analysis of the 12 resuscitated newborns found that early recognition and a rapid response, situational awareness, was critical for good clinical performance (9). These findings differed from our results with respect to specific skills as we found leadership, teamwork, and coping with stress and fatigue to underpin high clinical performance. Our interdisciplinary teams dealt with unplanned emergency situations in vaginally delivered newborns only, which may explain the differences in importance of specific skills. Interestingly, we found that coping with stress and communication with the parents was associated with high clinical performance score. We speculate that communication with parents may be a proxy variable also covering overview, self-confidence and clinical performance superiority, and communication with parents may not be prioritized if the team is coping poorly with stress and fatigue (33).

Clinical performance was in our study assessed by adherence to guidelines for neonatal resuscitation and transitional support. Recent studies have demonstrated that performance may fall short of expectations as only 20–25% of highly educated teams managed neonatal resuscitation with full adherence to the guideline (34, 35). In our study 51% of the teams achieved a high clinical performance defined by a score of 85–100, i.e., a rather different outcome. Evaluation of clinical performance in low resource settings in both Africa (36) and Nepal (37, 38) has also been conducted, however these studies differ from our study as they only evaluated video without any sound track.

Performance is likely to differ between centers (39). However, training of staff in the two hospitals included in the current study were led by the same team from Aarhus University Hospital, and all staff were trained using ERC guidelines and similar equipment. Still, there may be differences by staff and center, but our study sample failed to allow stratification by center or team composition.

Lower scores in our study were often related to delayed or no verbalization of the newborns heart rate and to the use of unnecessary CPAP according to the raters. Verbalization of the newborns heart rate is vital for the team performance, as the management depends on this information. In teams with lower scores the heart rate was often assessed late or not at all. Both hospitals trained all healthcare providers in neonatal resuscitation in simulation-based setup using a Laerdal Resusci baby®, which cannot simulate the heartrate. Therefore, the instructor provided this information by talking to the room as the “e.g., heartrate is 55” when the team asked for the heartrate. Hereby we may have introduced negative learning, as the teams never trained to assess the newborns heart rate. This must be addressed in the training programs. Furthermore, CPAP should only be given if medically indicated. In teams with lower scores, CPAP was often given without indication and for prolonged periods. Therefore, future training programs should focus both on when to initiate CPAP treatment and when to discontinue the treatment.

In the clinical setting, real life videos may be used for feedback, debriefing, quality assurance, and for identification of important focus areas for training programs or possible adjustments of training programs. For neonatal resuscitation, video has been used to identify the errors and inappropriate team behavior and to train teams to overcome these difficulties (40–42). However, this has never been shown to improve neonatal outcome (43). Another study including pediatric teams used video-based debriefing to improve adherence to guidelines (44) and found it lead to improved adherence to guidelines. Both studies investigated teams of pediatricians and future research should investigate how we can improve performance for interdisciplinary teams of midwives, obstetricians, anesthesiologists, and pediatricians on the delivery ward.

## Conclusion

By using video recordings in the delivery room, we assessed interdisciplinary *ad hoc* teams of midwives, obstetricians, anesthesiologists, and pediatricians and found that teams with an excellent non-technical score had a five times higher chance of high clinical performance compared to teams with an average non-technical score. We also found a dose-response like association between non-technical score and clinical performance. This was mainly driven by leadership and teamwork, coping with stress and fatigue. Future research should investigate how we can improve clinical performance and leadership, teamwork and coping strategies for stress and fatigue to ensure high clinical performance.

## Data Availability Statement

The raw data supporting the conclusions of this article will be made available by the authors, without undue reservation.

## Ethics Statement

The research project TeamOBS was approved legally and ethically in May 2014 by the Central Denmark Region, the Danish Data Protection Agency (2012-58-006) and the Research foundations of central Denmark (1-16-02-257-14). The patients/participants provided their written informed consent to participate in this study.

## Author Contributions

LB, LH, GE, NF, NU, OK, TH, and TM contributed to conception and design of the study. LB, LH, and OK contributed to data collection. LB, KH-H, TH, and GE assessed the videos. LB, NU, and TH contributed in data analysis. All authors contributed to the writing of this work. All authors contributed to the article and approved the submitted version.

## Funding

We thank the following organizations and departments for financial support: Tryg Foundation (Trygfonden) (Grant ID No. 109507); the Regional Hospital in Horsens, the Aarhus University Hospital, Department of Obstetrics and Gynecology; and the Regional Postgraduate Medical Education Administration Office North.

## Conflict of Interest

The authors declare that the research was conducted in the absence of any commercial or financial relationships that could be construed as a potential conflict of interest.

## Publisher's Note

All claims expressed in this article are solely those of the authors and do not necessarily represent those of their affiliated organizations, or those of the publisher, the editors and the reviewers. Any product that may be evaluated in this article, or claim that may be made by its manufacturer, is not guaranteed or endorsed by the publisher.
