# Supplementary material for: Teamwork and Adherence to Guideline on Newborn Resuscitation—Video Review of Neonatal Interdisciplinary Teams
Source: Front Pediatr. 2022 Feb 21;10:828297. doi: 10.3389/fped.2022.828297 (PMC8900704; doi:10.3389/fped.2022.828297)
Supplement: Supplementary file 1 [file Data_Sheet_1.pdf]

Supplementary Material

TEAM | OBS

Clinical Performance Checklist evaluating newborn resuscitation and support of transition of infants at birth *Scoring (Put a cross in the box; and give 0, 1 or 2 points, as appropriate)*

Stage of management	Item No.	Item description	Not indicated/ cannot be resist	Not done or incorrectly done	Partially or not done in a timely manner	Done correctly, completely and in a timely manner	Weighted score
				0 points	1 point	2 points	
Preparation of the room	1-1	Sets up the Neopuff (FiO2 .21, peep 5, peak 30, flow 8-10 L)	☐	☐	Safe, but could be improved ☐	Immediate accessable ☐	
	1-2	Bag/valve mask	☐	☐	Safe, but could be improved ☐	Immediate accessable ☐	
	1-3	Suction set at 80-100 mmHg and catheters available	☐	☐	Safe, but could be improved ☐	Immediate accessable ☐	
	1-4	Radiant warmer on	☐	☐	Safe, but could be improved ☐	Immediate accessable ☐	
	1-5	Linen, hat and blankets	☐	☐	Safe, but could be improved ☐	Immediate accessable ☐	
	1-6	Stethoscope	☐	☐	Safe, but could be improved ☐	Immediate accessable ☐	
	1-7	Clock	☐	☐	Safe, but could be improved ☐	Immediate accessable ☐	
	1-8	Pulse oximeter	☐	☐	Safe, but could be improved ☐	Immediate accessable ☐	
	1-9	Respiratory Therapy - Intubation equipment checked (Laryngoscope, blade, tubes, CO2 sensor)	☐	☐	Safe, but could be improved ☐	Immediate accessable ☐	
Initial assessment and support of transition	2-1	Dry neonate and remove wet linen	☐	☐	Safe, but could be improved ☐	Managed according to guideline ☐	
	2-2	Place neonate correctly with neutral position	☐	☐	Safe, but could be improved ☐	Managed according to guideline ☐	
	2-3	Assess Airway, Breathing, color and tone	☐	☐	Safe, but could be improved ☐	Managed according to guideline ☐	
	2-4	Assess Heartrate	☐	☐	Safe, but could be improved ☐	Managed according to guideline ☐	
	2-5	Suction only if indicated	☐	☐	Safe, but could be improved ☐	Managed according to guideline ☐	
	2-6	No breathing or gasping – 5 inflation breaths (2-3 seconds per breath)	☐	☐	Safe, but could be improved ☐	Managed according to guideline ☐	
	2-7	Reassess breathing, heartrate and tone	☐	☐	Safe, but could be improved ☐	Managed according to guideline ☐	
A-problem	3-1	Recheck head position	☐	☐	Safe, but could be improved ☐	Managed according to guideline ☐	
	3-2	Secure patent airway (oropharyngeal airway or suction)	☐	☐	Safe, but could be improved ☐	Managed according to guideline ☐	
	3-3	Positive pressure ventilation	☐	☐	Safe, but could be improved ☐	Managed according to guideline ☐	
	3-4	Intubation	☐	☐	Safe, but could be improved ☐	Managed according to guideline ☐	
	3-5	Expiratorisk CO2 sensor	☐	☐	Safe, but could be improved ☐	Managed according to guideline ☐	
B-problem	4-1	Heart rate <100: ventilate	☐	☐	Safe, but could be improved ☐	Managed according to guideline ☐	
	4-2	Not breathing and heartrate >100: ventilate 30-40 /minutes	☐	☐	Safe, but could be improved ☐	Managed according to guideline ☐	
	4-3	Breathing irregular and heartrate >100: consider ventilation	☐	☐	Safe, but could be improved ☐	Managed according to guideline ☐	
	4-4	Increased work of breathing and heart rate >100: consider CPAP	☐	☐	Safe, but could be improved ☐	Managed according to guideline ☐	
C-problem	5-1	Heart rate <60: increase FiO2	☐	☐	Safe, but could be improved ☐	Managed according to guideline ☐	
	5-2	Heartrate <60: start chest compression (3 compressions to 1 breath)	☐	☐	Safe, but could be improved ☐	Managed according to guideline ☐	
	5-3	Reassess heart rate every 30 seconds	☐	☐	Safe, but could be improved ☐	Managed according to guideline ☐	
	5-4	Vascular access and adrenalin, fluid or blood	☐	☐	Safe, but could be improved ☐	Managed according to guideline ☐	
Total			sum				sum
Patient safety score							

0% — 50% — 100%

Patient safety is threatened/ Non-acceptable performance | Acceptable performance | Excellent

% = Patient safety score

Weighted score: (___ [sum] / 58 − ___ [sum]) x 100 = ___

Clinic performance score
(patient safety score + weighted score / 2 = ___

Grafisk Service 6401

Supplemental Figure 1
The checklist TeamOBS-newborn for assessment of adherence to ERC guideline for neonatal resuscitation and transitional support

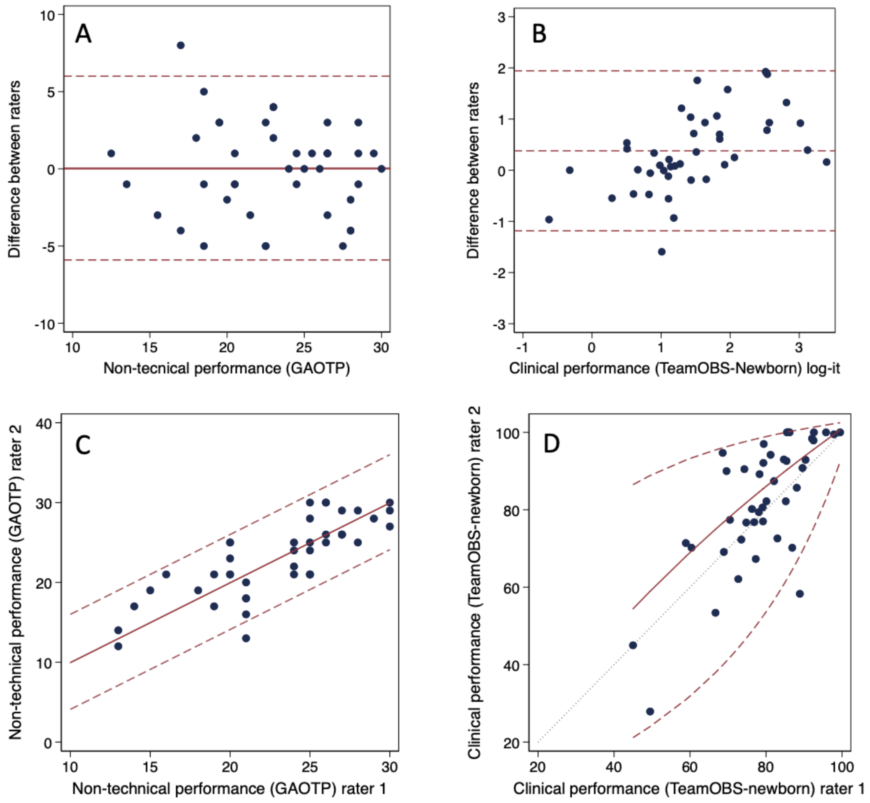

Supplemental Figure 2

Bland Altman plots and limits of agreement (A+B). Clinical performance was analysed on the log-it scale to meet the assumptions of constant mean, SD and normality. Interrater agreement visualized as function of rater 1 and rater 2 (C+D). The 95% confidence interval are curved in graph D as it was transformed from log-it sale (B) to original scale (D)

Supplemental Table 1. Inter-rater agreement for non-technical score on the item level for GAOTP score

	Descriptive		Kappa	Difference (%) *				
GAOTP Items	Mean	Range	Weighted	dif=0	dif=1	dif=2	dif=3	dif=4
Item 1 Communication with patient	4.2	(1-5)	0.85	0.50	0.38	0.12	0.00	0.00
Item 2 Task/case management	3.6	(1-5)	0.86	0.62	0.24	0.12	0.02	0.00
Item 3 Teamwork	3.4	(1-5)	0.82	0.43	0.43	0.14	0.00	0.00
Item 4 Situational awareness	4.2	(1-5)	0.90	0.74	0.14	0.12	0.00	0.00
Item 5 communication with team members	3.5	(1-5)	0.80	0.45	0.34	0.19	0.00	0.02
Item 6 Environment of the room	4.1	(1-5)	0.86	0.55	0.38	0.05	0.02	0.00

* Difference in percentage: for example, the raters agreed perfectly with a difference of 0 on item 1.1 in 80% of the

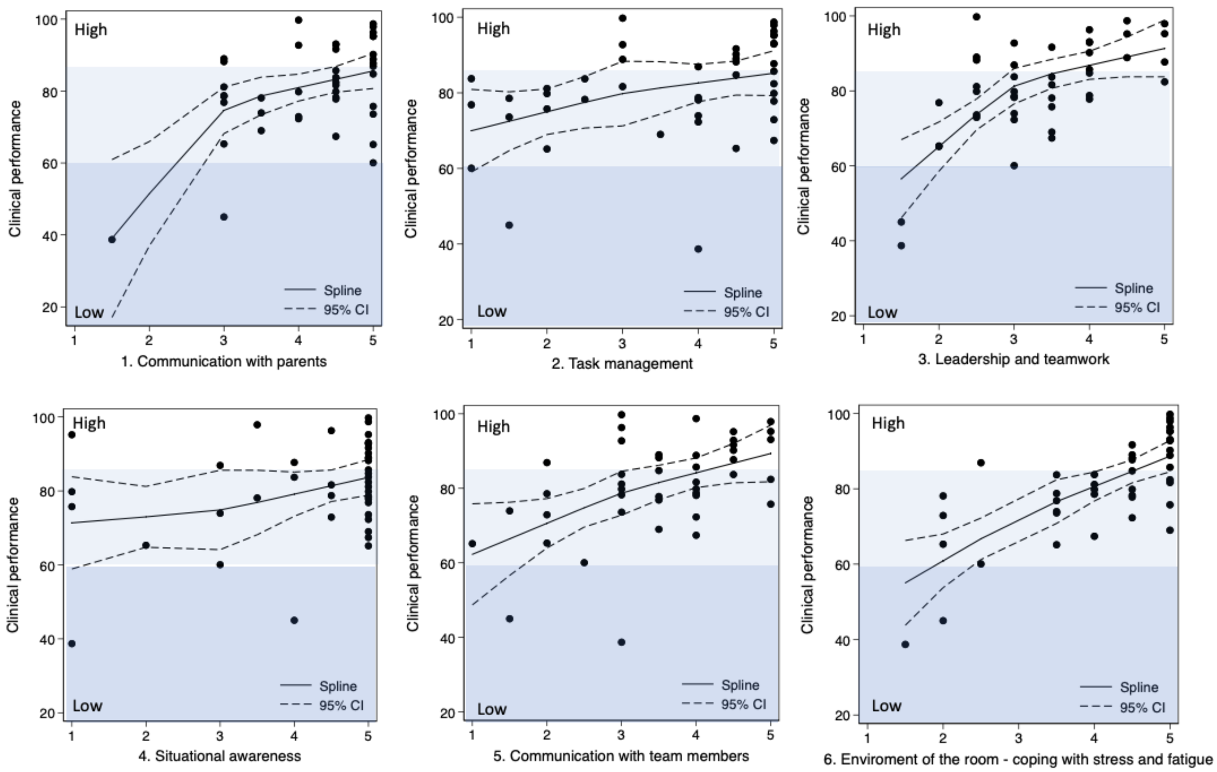

Supplemental Figure 3
The association between clinical performance and the non-technical skills on item level.
